# Next-generation sequencing reveals somatic mutations that confer exceptional response to everolimus

**DOI:** 10.18632/oncotarget.7234

**Published:** 2016-02-07

**Authors:** Sun Min Lim, Hyung Soon Park, Sangwoo Kim, Sora Kim, Siraj M. Ali, Joel R. Greenbowe, In Seok Yang, Nak-Jung Kwon, Jae Lyun Lee, Min-Hee Ryu, Jin-Hee Ahn, Jeeyun Lee, Min Goo Lee, Hyo Song Kim, Hyunki Kim, Hye Ryun Kim, Yong Wha Moon, Hyun Cheol Chung, Joo-Hang Kim, Yoon-Koo Kang, Byoung Chul Cho

**Affiliations:** ^1^ Division of Medical Oncology, Department of Internal Medicine, Yonsei University College of Medicine, Seoul, Korea; ^2^ Division of Medical Oncology, Department of Internal Medicine, CHA Bundang Medical Center, CHA University, Seongnam, Korea; ^3^ Department of Pharmacology and Brain Korea 21 Plus Project for Medical Sciences, Yonsei University College of Medicine, Seoul, Korea; ^4^ Severance Biomedical Science Institute and Brain Korea 21 Plus Project for Medical Sciences, Yonsei University College of Medicine, Seoul, Korea; ^5^ Foundation Medicine Inc, Cambridge, MA, USA; ^6^ MacroGen Inc., Seoul, Korea; ^7^ Department of Oncology, University of Ulsan College of Medicine, Asan Medical Center, Seoul, Korea; ^8^ Division of Hematology-Oncology, Department of Medicine, Samsung Medical Center, Sungkyunkwan University School of Medicine, Seoul, Korea; ^9^ Department of Pathology, Yonsei University College of Medicine, Seoul, Korea

**Keywords:** everolimus, NF1, TSC1, mTOR, next-generation sequencing

## Abstract

**Background:**

Given the modest responses to everolimus, a mTOR inhibitor, in multiple tumor types, there is a pressing need to identify predictive biomarkers for this drug. Using targeted ultra-deep sequencing, we aimed to explore genomic alterations that confer extreme sensitivity to everolimus.

**Results:**

We collected formalin-fixed paraffin-embedded tumor/normal pairs from 39 patients (22 with exceptional clinical benefit, 17 with no clinical benefit) who were treated with everolimus across various tumor types (13 gastric cancers, 15 renal cell carcinomas, 2 thyroid cancers, 2 head and neck cancer, and 7 sarcomas). Ion AmpliSeq^TM^ Comprehensive Cancer Panel was used to identify alterations across all exons of 409 target genes. Tumors were sequenced to a median coverage of 552x. Cancer genomes are characterized by 219 somatic single-nucleotide variants (181 missense, 9 nonsense, 7 splice-site) and 22 frameshift insertions/deletions, with a median of 2.1 mutations per Mb (0 to 12.4 mutations per Mb). Overall, genomic alterations with activating effect on mTOR signaling were identified in 10 of 22 (45%) patients with clinical benefit and these include *MTOR, TSC1, TSC2, NF1*, *PIK3CA* and *PIK3CG* mutations. Recurrently mutated genes in chromatin remodeling genes (*BAP1; n* = 2, 12%) and receptor tyrosine kinase signaling (*FGFR4; n* = 2, 12%) were noted only in patients without clinical benefit.

**Conclusions:**

Regardless of different cancer types, mTOR-pathway-activating mutations confer sensitivity to everolimus. Targeted sequencing of mTOR pathway genes facilitates identification of potential candidates for mTOR inhibitors.

## BACKGROUND

Mammalian target of rapamycin (mTOR) is a key component of phosphatidylinositol 3-kinase (PI3K) pathway that plays an important role of cell growth and proliferation, metabolism, and angiogenesis [1]. Since inhibition of mTOR signaling can abrogate the cellular response to growth factor receptor activation, targeting mTOR activation is an attractive approach for cancer therapy.

Everolimus is a rapamycin analog that is being developed as an inhibitor of mTORC1. Like rapamycin, everolimus binds the cyclophilin FKBP-12, and this complex binds the serine-threonine kinase of mTORC1 and inhibits signaling downstream. Everolimus has been extensively tested for several kinds of tumor types, and showed some significant and durable responses. In renal cell carcinoma, everolimus provided a significant benefit to patients with metastatic renal cell carcinoma after failure of treatment with sorafenib or sunitinib [2]. Adding everolimus to exemestane significantly improved median progression-free survival (PFS) compared with exemestane alone in the treatment of estrogen receptor-positive, HER2-negative advanced breast cancer patients [3]. In addition, everolimus provided a significant reduction in volume and seizure frequency in patients with subependymal giant cell astrocytoma associated with tuberous sclerosis, and prolonged survival in pancreatic neuroendocrine tumor [4, 5].

However, there has not been a validated biomarker for predicting response to everolimus, yet. Previous studies used candidate gene approach for searching mutations and revealed that PI3K/AKT/mTOR pathway had an important role in everolimus sensitivity [6-8]. Recently, whole genome sequencing identified a somatic mutation for everolimus sensitivity in bladder cancer [9]. In this study, mutation of *tuberous sclerosis 1* (*TSC1*) was suggested as a biomarker for everolimus response in bladder cancer. Similarly, one anaplastic thyroid cancer patient who showed sensitivity to everolimus, revealed a nonsense mutation in *TSC2*, a negative regulator of mTOR [10]. A recent study by Yoon *et al*. reported that pS6^Ser240/4^ expression may be a predictive biomarker for everolimus sensitivity in gastric cancer patients [11]. Likewise, we recently discovered one patient who showed exceptional response to everolimus.

We hypothesized that there are driver genetic events, which are clinically actionable and targetable, that occur commonly across different tumor types. In this study, we aim to perform next-generation sequencing (NGS) in patients with different tumor types to explore common genetic aberrations that confer sensitivity to everolimus. We recruited patients who were treated with single-agent everolimus, and sought to find universal biomarkers regardless of tumor types and histologic subtypes.

## RESULTS

### Everolimus leading to response in a patient with primary ductal adenocarcinoma of the lacrimal gland

**Figure 1:**
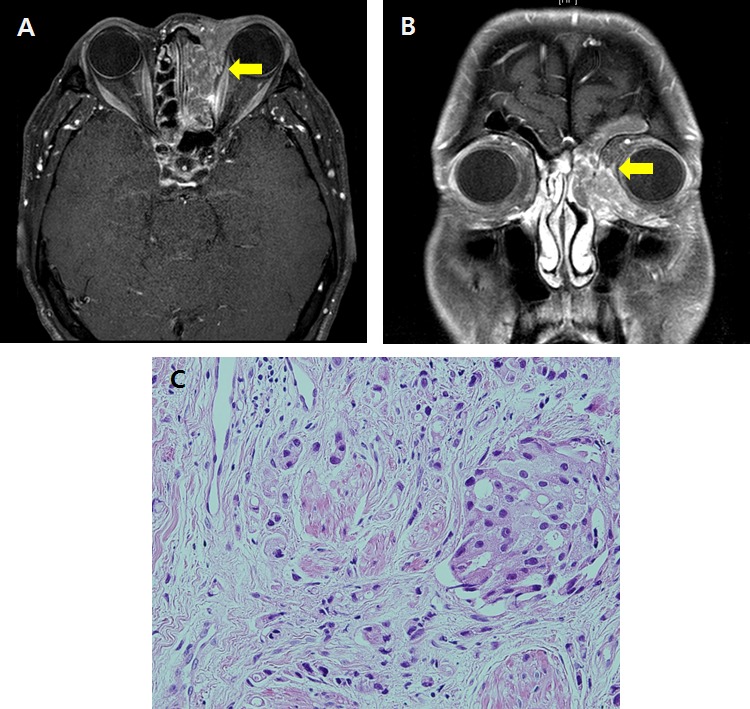
The primary tumor located in the lacrimal gland seen in orbit MRI **A.** axial view **B.** coronal view. **C.** Hematoxylin & eosin staining of the tumor.

Our index patient was a 51-year-old male who diagnosed with primary ductal adenocarcinoma of the lacrimal gland in the left. (Figure 1). The patient received multiple lines of chemotherapy, but patient showed aggravation of primary tumor and progression to bone metastasis. His tumor tissue was sent for targeted next-generation sequencing at Foundation Medicine profiling of 236 cancer-related genes and 47 introns of 19 genes involved in rearrangement. The assay reported *TP53* (Q38fs) mutation and *NF1* (D1644A) mutation (Supplementary Figure S2A-B). While there were no approved therapies or clinical trials to address for *TP53* mutation and *NF1* mutation at that time, a therapeutic attempt using everolimus 10mg once per day was initiated in October 2013 on the basis of data published by McGillicuddy *et al*, supporting the sensitivity of everolimus in loss-of-function mutation in the *NF1* tumor suppressor gene [12]. The patient was seen again 1 month after, and we noticed decreased exophthalmus and reduced skin thickening around his left eyelid. In terms of subjective symptoms, he reported improved pain of his bone metastasis lesions in right humerus head and left shoulder. The PET-CT taken after 1 month showed a partial response as per PET response criteria [13], showing more than 25% reduction of SUV_max_ compared to baseline. He was maintained on everolimus and the CT scan taken after 2 months of therapy showed shrinkage of the measurable tumor lesions (26.5mm to 17.6mm, 33.5% reduction). His tumor showed further decrease in SUV_max_ in the PET-CT scan taken after 4 months of therapy (Supplementary Figure 3). The partial response was maintained for 8 months.

### Patients' characteristics

We collected a total of thirty-nine patients with five different tumor types (13 with gastric cancer, 15 with renal cell carcinoma, 2 with thyroid cancer, 2 with head and neck cancer, and 7 with sarcoma) were analyzed by NGS. As shown in Table 1, the median age of all patients was 57, and there were 24 (61.5%) males and 15 (38.5%) females. There were 22 (56.4%) patients with clinical benefit and 17 (43.6%) patients without clinical benefit. Patients with clinical benefit included 9 patients with partial response (PR) and 13 patients with durable stable disease (SD), with 10 patients showing SD ≥ 6 months. All patients without clinical benefit showed progressive disease (PD) as their best response with no tumor shrinkage at all. The median progression-free survival was 13.0 months (95% CI, 6.3-19.7) for patients with clinical benefit and 1.7 months (95% CI, 1.54-1.85) for patients without clinical benefit. As of data cutoff, two patients were still under ongoing everolimus treatment (Supplementary Table 3, 4).

**Table 1:** Baseline characteristics of all patients (n=39)

Characteristics	No.	%
Sex		
Male	24	62
Female	15	38
Age		
Median age, years (range)	57 (23-83)	
Tumor type		
Gastric cancer	13	33
Renal cell carcinoma	15	38
Clear-cell carcinoma	15	38
Non-clear-cell carcinoma	0	0
Thyroid cancer	2	4
Anaplastic thyroid cancer	1	2
Medullary thyroid cancer	1	2
Head and Neck cancer^1^	2	5
Sarcoma	7	17
Angiosarcoma	2	5
Malignant fibrous histiocytoma	2	5
Leiomyosarcoma	2	5
Fibrosarcoma	1	2
Patient group		
Clinical benefit	22	56
Non-clinical benefit	17	44
Tumor response		
CR	0	0
PR	9	23
SD^2^	13	33
PD^3^	17	44
Median progression-free survival (mo, 95% CI)		
Clinical benefit	13.0 (6.3-19.7)	
Non-clinical benefit	1.7 (1.5-1.8)	

1 One patient was diagnosed with primary ductal carcinoma of the lacrimal gland, and the other patient was diagnosed with carcinosarcoma of parotid gland

2 Of 13 SD patients, 10 patients showed SD ≥ 6 months.

3 All non-responders had progressive disease as best response

4 As of June, 2015, 2 patients were ongoing everolimus.

### Overall genetic alterations

To identify possible genetic mechanisms of sensitivity to everolimus, we performed targeted sequencing of the pre-treatment tumor and paired germline DNA. Tumors were sequenced to a median coverage of 552x. Cancer genomes were characterized by 219 somatic single-nucleotide variants (181 missense, 9 nonsense, 7 splice-site) and 22 frameshift insertions/deletions, with a median of 2.1 mutations per Mb (0 to 12.4 mutations per Mb). Of these alterations, 13 mutations were previously reported in the Catalogue of Somatic Mutations in Cancer. Mutations were further analyzed for functional prediction of amino acid changes using two different prediction algorithms (Provean and SIFT) (Supplementary Table 5). *TP53* gene alterations were the most common among all genes and were found in 8 of 38 tumors (21.1%). Recurrently mutated genes such as *PBRM1, MTOR, NF1, VHL, PD34DIP, and ARID1A* were found in patients with clinical benefit, in the order of frequency. In contrast, recurrently mutated genes such as *VHL, TP53,* and *ARID1A* were found in patients with non-clinical benefit. (Supplementary Figure S4). Sanger sequencing for mTOR pathway genes (*MTOR/TSC1/TSC2/PIK3CA/NF1/NF2/PTEN/AKT1*) confirmed variants that were found in CCP panel (supplementary Figure S5 & supplementary Table 6).

### Pathways relevant to everolimus sensitivity

**Figure 2:**
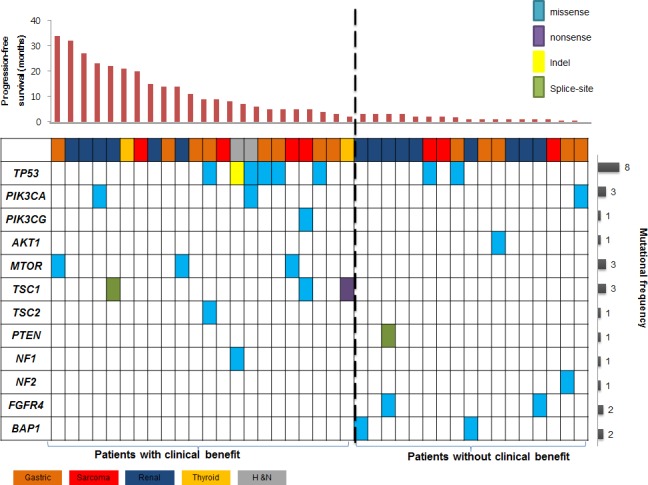
Landscape genomic profile of patients is seen Genomic alterations which may activate mTOR signaling were identified in 10 of 22 (45%) patients with clinical benefit. Recurrently mutated genes of *FGFR4* and *BAP1* were noted only in patients without clinical benefit (*P* = NS).

We examined the sequencing data for biologically plausible mechanisms of sensitivity to everolimus, and identified multiple mutations in the mTOR-pathway (Figure 2).

Two *PIK3CA* mutations were detected in patients with clinical benefit. A mutation in the helical domain of *PIK3CA* (p.E542K), which is known to be constitutively activating and selectively sensitive to everolimus [14], was found in a renal cell carcinoma patient with the PFS of 23.9 months. In addition, a kinase domain mutation of *PIK3CA* (p.H1047R) was found in a head and neck cancer patient with partial response. Alterations in *TSC1* gene were noted in three patients: a patient with anaplastic thyroid cancer who harbored a nonsense mutation in *TSC1* (p.Trp103*) and a renal cell carcinoma patient with a *TSC1* splicing variant (c.1029+1G>A). In addition, a malignant histiosarcoma patient had *TSC1* missense mutation (p.A307V) and showed significant tumor reduction (−24.3%). One *TSC2* missense mutation (p.E588K) was found in a gastric cancer patient. Three *MTOR* missense mutations (N1421D, K1771R, I1973F) were found in patients with gastric cancer, angiosarcoma and renal cell carcinoma, respectively. All three mutations were located in FAT (FRAP, ATM, TRAP) domain of MTOR.

**Figure 3:**
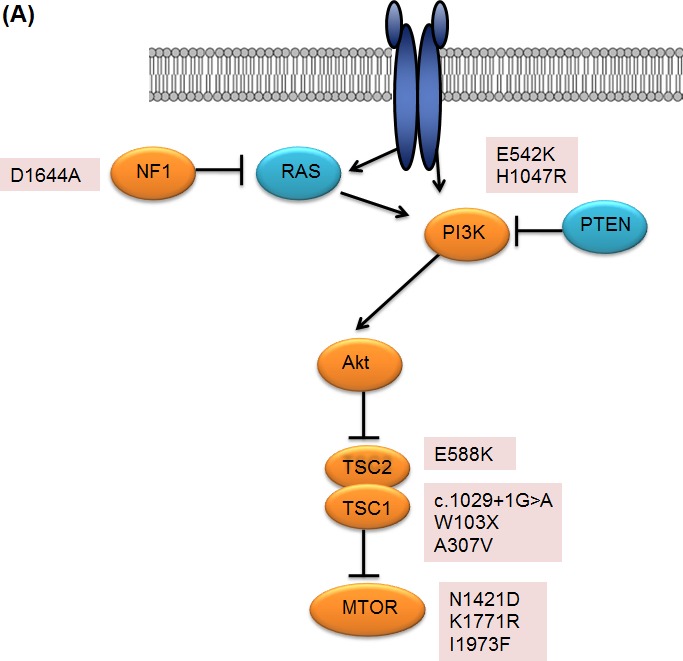

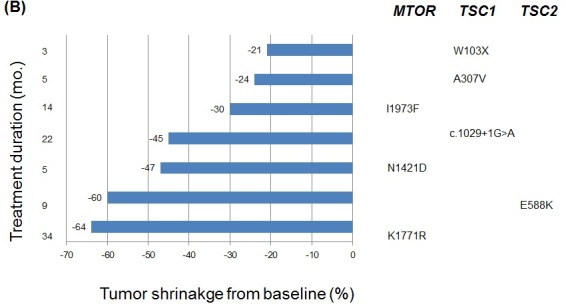
**A.** Genetic alterations identified in patients with clinical benefit **B.** Best overall response of patients with clinical benefit, with negative values indicating tumor shrinkage and the vertical axis indicating treatment duration in months. Nonsynonymous mutations for each patient are marked accordingly.

Overall, genomic alterations which may activate mTOR signaling were identified in 10 of 22 (45%) patients with clinical benefit (Figure 3A). In particular, *TSC1/TSC2/MTOR* mutations were key components in determining everolimus sensitivity (Figure 3B). The incidence of these mutations were 31.8% (7/22) in patients with clinical benefit as compared with 0% in those with non-clinical benefit (*P*=0.012). The prevalence of these recurrently mutated genes and their correlation with clinical benefit strongly suggest that they confer sensitivity to everolimus.

While *TSC1/TSC2/MTOR* alterations were exclusively found in patients with clinical benefit, we also searched for recurrently mutated genes that were exclusively identified in patients with non-clinical benefit. Mutations in chromatin remodeling gene (*BAP1;* n=2, 12%) and receptor tyrosine kinase signaling (*FGFR4;* n=2, 12%) were noted, but not statistically significant.

## DISCUSSION

Here, we described multiple activating mutations in the mTOR pathway found in patient tumors that showed exquisite sensitivity to everolimus. *TSC1/TSC2/MTOR* alterations were exclusively found in patients who showed extended clinical benefit, which suggest that they confer sensitivity to everolimus. *NF1* mutation together with *TP53* mutation presents an aggressive tumor behavior, but tumor growth is effectively inhibited by everolimus.

In theory, dependency on the *mTOR* pathway may render enhanced sensitivity to mTOR inhibitors. Three different mutations (N1421D, K1771R, I1973F) in the FAT domain of *MTOR* have not been previously reported in the publicly available genomic database [15]. Previously, it has been proposed that FAT domain consist of TRD1, TRD2 and TRD3 domain and interacts to yield a configuration that exposes the catalytic domain [16, 17]. Especially, N1421D was located in TRD1 domain which is important for mTOR function and both Provean and SIFT predicted that our mutation was deleterious mutation. In addition, K1771R mutation is located in TRD3 which is interact with the C lobe on one side of the kinase domain. Mutation of I1973 amino acid was previously reported to be hyperactivating mutation which involved in restricting active site access and both Provean and SIFT reported that I1973F is deleterious mutation [17].

TSC1 and TSC2 are upstream key negative regulators of mTORC1. They form a heterotrimer, a GTPase-activating protein for Rheb (Ras homologue enriched in brain), a GTP-binding protein that activates mTORC1 by binding to it. As the loss of TSC1-TSC2 function activates mTORC1 pathway, strong scientific rationale exists for the use of mTOR inhibitors in this setting. Sirolimus has shown promising effects in patients with inactivating mutations in *TSC1*, *TSC2* and *STK11* in hamartoma syndromes such as tuberous sclerosis complex and Peutz-Jeghers Syndrome [18-20]. Moreover, 2 out of 3 patients with malignant perivascular epithelioid cell tumors who had clinical response to sirolimus showed *TSC1/2* loss [21]. There was a recent report of renal cell carcinoma patients with extended benefit from mTOR inhibitor showed that *TSC1* and *TSC2* offer explanation for treatment response [22]. Similarly, we identified 1 *TSC1* splicing variant (renal cell carcinoma), 1 *TSC1* nonsense mutation (anaplastic thyroid cancer), 1 *TSC1* missense mutation (sarcoma) and 1 *TSC2* missense mutation (angiosarcoma). Given that heterozygous loss of *TSC1* is common in renal cell carcinoma (>30%) [23], *TSC1* and *TSC2* may be screened as predictive biomarkers of everolimus in renal cell carcinoma patients who progressed on VEGF-targeted therapy.

Loss-of-function mutations in the *NF1* tumor suppressor gene affects mTOR pathway, leading to constitutive activation of mTOR. This activation depends on Ras and PI3 kinase, and is mediated by the inactivation of the *TSC2*-encoded protein tuberin by AKT [24]. In this way, *NF1*-deficient tumors alone may show exquisite sensitivity to mTOR inhibitors. In conjunction with our index patient with *TP53* and *NF1* mutation, it has been reported that *NF1* heterozygosity cooperates with *TP53* mutation to promote tumorigenesis. Homozygous null *NF1* mutations may occur, which mediate sensitivity to mTOR inhibitors [12]. Although rare in tumors such as gastric cancer, sarcoma, and renal cell carcinoma, recent finding that *NF1* mutations are enriched (11%) in oncogene-negative subset of lung adenocarcinomas, and our finding suggests novel therapeutic opportunities for the subset of patients with *NF1* mutations.

Conceptually, activating mutations within the phosphoinositide 3-kinase, catalytic subunit α (PI3K-p110 α) encoded by the *PIK3CA* gene, lead to mTOR pathway activation [6, 25]. In this study, activating *PIK3CA* mutations in helical domain (p.E542K), and kinase domain (p.H1047R) were found in a renal cell carcinoma and a parotid gland choriocarcinoma patient, respectively. However, one patient with a *PIK3CA* mutation (p.H1047R) showed non-clinical benefit to everolimus, showing rapid increase of ascites within 1 month of everolimus treatment. That activating *PIK3CA* mutation does not always lead to response was previously reported by Janku et al. where they described patients with H1047R *PIK3CA* mutation experienced a response rate of 44% [26].

Our study has a few limitations. Although prespecified, the selected patients with clinical benefit in this study may only explain a portion of mechanisms of sensitivity. Other genetic or epigenetic alterations that are not covered in the CCP may be missed out even if they regulate sensitivity or resistance to everolimus. Because study was retrospective in nature, and the number of patients analyzed was relatively small, these data must be interpreted cautiously

However, to the best of our knowledge, this is the first report that identified histology-independent mechanisms of sensitivity to mTOR inhibitor in multiple tumor types. Regardless of histology, mTOR pathway-activating mutations confer exceptional response to everolimus, and this provides the rationale for the development of so-called basket trials. The current study highlights the fact that mutational analyses of somatic variants could allow sub-classification of patients for optimal treatment, and provides a basis for a basket trial in the future. These efforts will eventually increase the likelihood of success of drug trials especially in patients with rare cancer types.

In conclusion, the genomic information derived from patients with exceptional clinical benefit may provide a basis for everolimus sensitivity. Overall, it seems that screening for *TSC1/TSC2/MTOR* mutations warrants further investigation in application of mTOR inhibitors in the clinic.

## MATERIALS AND METHODS

### Patients

We recruited tissue samples and matched blood samples among patients who participated in the clinical trials using everolimus and renal cell carcinoma patients treated with everolimus as a standard second line of therapy (Supplementary Table 1). Patients with extended clinical benefit and non-benefit were selected for this study. The criteria for extended clinical benefit were: 1) complete response or 2) partial response for more than 6 months or 3) durable stable disease with PFS on everolimus ≥ 1.5 x PFS of prior treatment. The criteria for non-benefit were 1) no shrinkage in tumor diameter and 2) progressive disease as best response.

Clinical information including age, sex, treatment duration, best response to treatment, percent change in tumor size, previous treatment history and survival data were collected. The study protocol was approved by each center's independent ethics committee or institutional review board and was conducted in accordance with the Declaration of Helsinki and Good Clinical Practice. All patients provided written informed consent for genomic testing used for this study. Specimens were evaluated by pathologists (H.K.K) to identify tumor-bearing areas for DNA extraction.

### Targeted sequencing

Tumor genomic DNA was extracted from formalin-fixed, paraffin-embedded (FFPE) tumor tissue blocks, unstained 10 um thick tissue sections. All samples were micro-dissected to ensure ≥70% tumor contents. Normal genomic DNA was extracted from either peripheral blood mononuclear cells or histologically confirmed normal tissue. Library preparation for each sample was performed using Ion AmpliSeq^TM^ Comprehensive Cancer Panel (CCP, Life Technology) following the manufacturer's instructions. The pooled capture library was quantified by Qubit (Invitrogen) and Tape station (Agilent) and sequenced by Ion Proton^TM^ System. Two patients (anaplastic thyroid cancer and renal cell carcinoma) were sequenced by Sanger sequencing using primers for all coding region of *TSC1*.

### Analysis of molecular aberrations

Ion AmpliSeq™ *Comprehensive Cancer Panel* (CCP, Life Technologies) was used for cancer specific target sequencing, targeting 409 genes with ∼16,000 amplicons. Sequencing was processed by Ion PGM^TM^ system and sequencing data was analyzed by PGM^TM^ built in TSS (Torrent Suite *Software*) 4.0.2 version (Supplementary Fig S1). Variants acquired from the CCP panel were filtered by germline variants acquired from our patients and 1000 genome data. Mutations with less than 50x depth and less than 10% variant frequency were filtered out [27]. Quality score, a parameter of variant call format using phred scale, was used to filter out the variants and Q30 was used for cut-off value [27]. Then, variants were annotated using ANNOVAR [28] and non-coding region and synonymous variants were filtered out. Remaining variants were assessed using the Integrative Genomics Viewer (IGV) [29] and loci were further analyzed for functional prediction of amino acid changes using two different prediction algorithms (Provean and SIFT) [30, 31]. Mutations in *MTOR, TSC1, TSC2, PIK3CA, NF1, NF2, PTEN, AKT1* were validated by Sanger sequencing or pyrosequencing. Sanger sequencing and pyrosequencing primers listed in the supplementary table 2.

### Statistical analyses

Tumor response was evaluated using the RECIST v1.1 [32] and progression-free survival (PFS) was defined as the time from the start of everolimus to disease progression or death from any cause. Statistical significance of preferential association of somatic variations with specific clusters was assessed with chi-square test or Fisher's exact test. All statistical analyses were performed using SPSS 20.0 (SPSS, Chicago, IL, USA).

## SUPPLEMENTARY MATERIAL FIGURES AND TABLES





## Abbreviations

PFS: progression-free survival
NGS: next-generation sequencing
FFPE: formalin-fixed, paraffin-embedded
CCP: Comprehensive Cancer Panel
IGV: Integrative Genomics Viewer
RECIST: Response evaluation criteria in solid tumor
PR: partial response
SD: stable disease
PD: progressive disease
