# Supplementary material for: Next-generation sequencing reveals somatic mutations that confer exceptional response to everolimus
Source: Oncotarget. 2016 Feb 7;7(9):10547–56. doi: 10.18632/oncotarget.7234 (PMC4891139; doi:10.18632/oncotarget.7234)
Supplement: Supplementary file 1 [file oncotarget-07-10547-s001.pdf]

# Next-generation sequencing reveals somatic mutations that confer exceptional response to everolimus

**Supplementary Material**

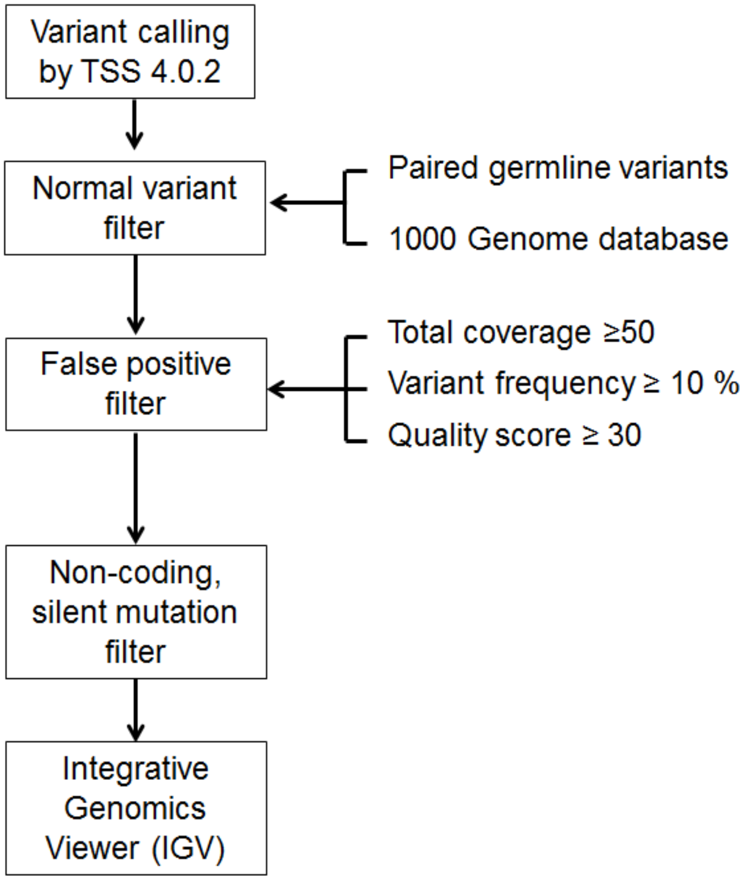


**Supplementary Fig.S1.** Variants acquired from the CCP panel were filtered by germline variants acquired from our patients and 1000 genome data. Mutations with less than 50x depth and less than 10% variant frequency were filtered out. Quality score, a parameter of variant call format (VCF) using phred scale, was used to filter out the variants and Q30 was used for cut-off value. Then, variants were annotated using ANNOVAR and non-coding region and synonymous variants were filtered out.

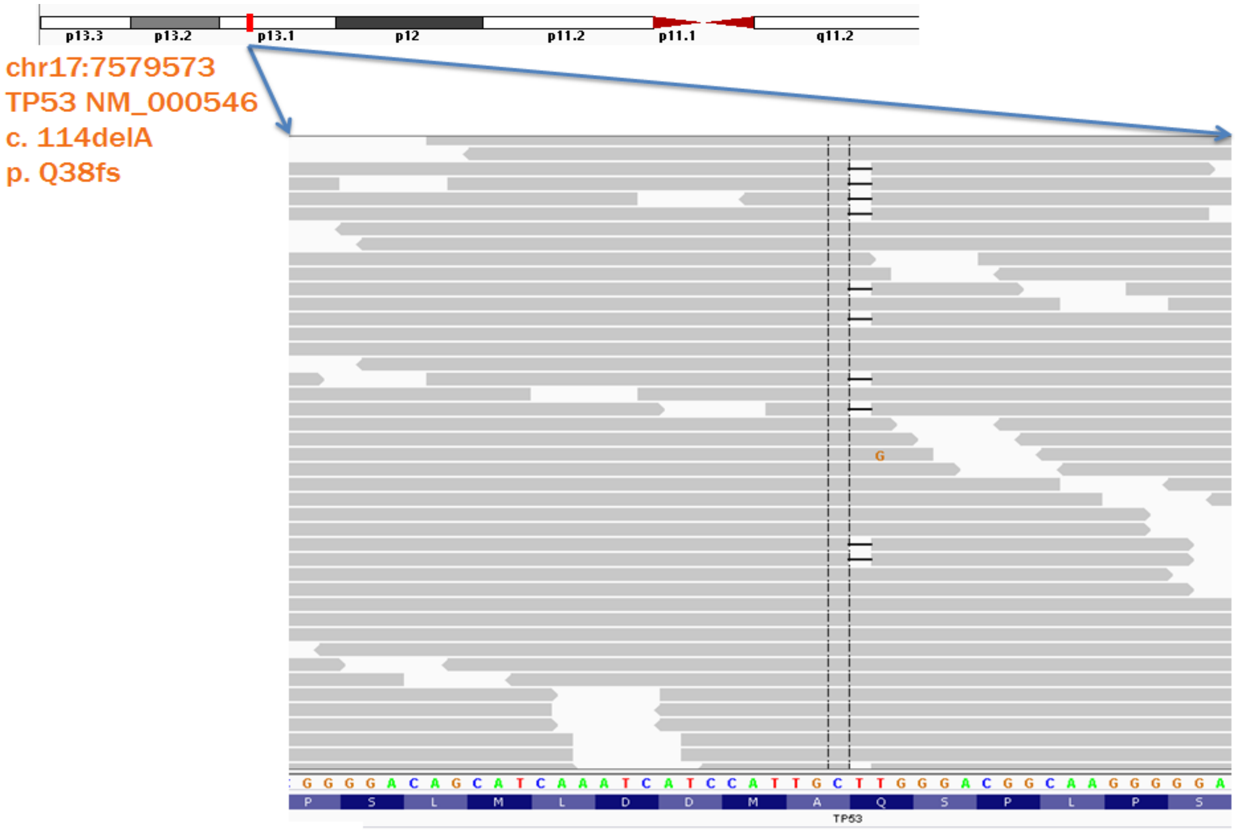


**(A)** *TP53* (Q38fs) mutation in Integrative Genomics Viewer

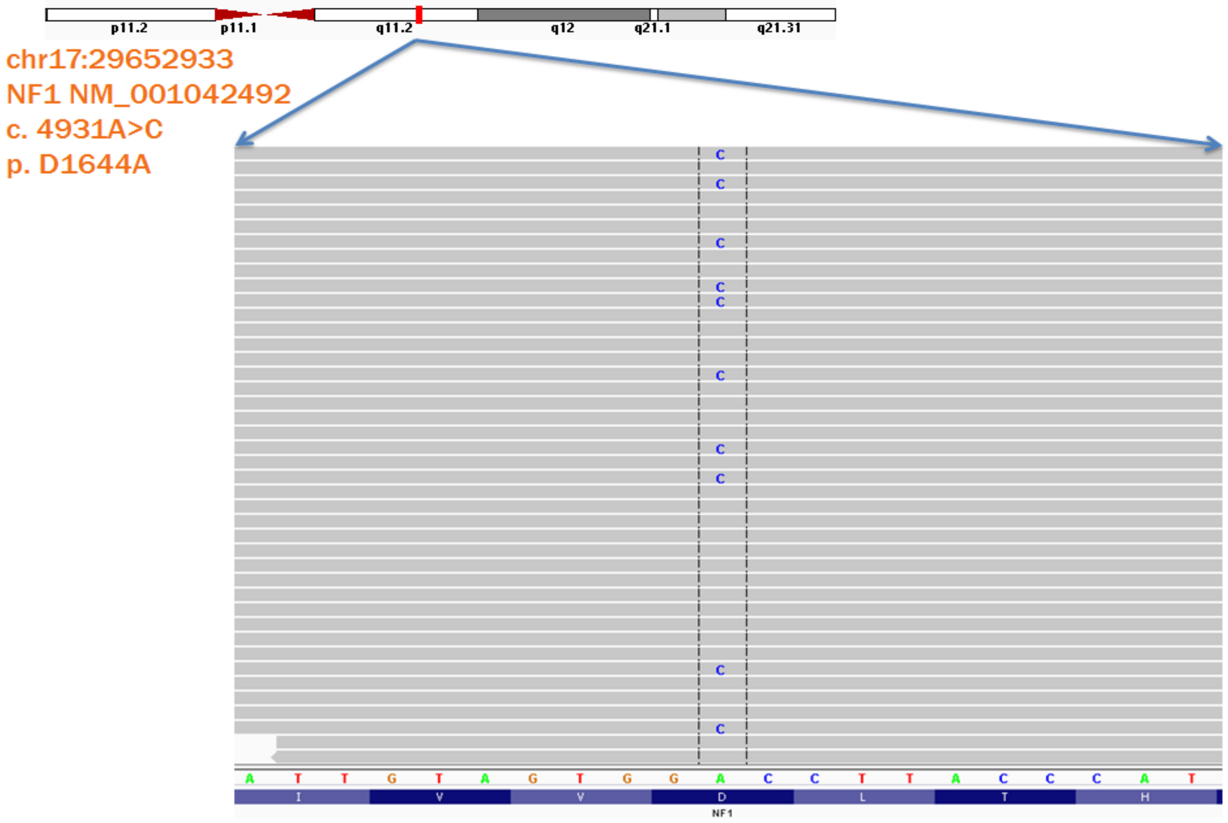


**(B).** *NF1* (D1644A) mutation in Integrative Genomics Viewer

**Supplementary Fig.S2. (A)** *TP53* (Q38fs) mutation and **(B)** *NF1* (D1644A) mutation in Integrative Genomics Viewer

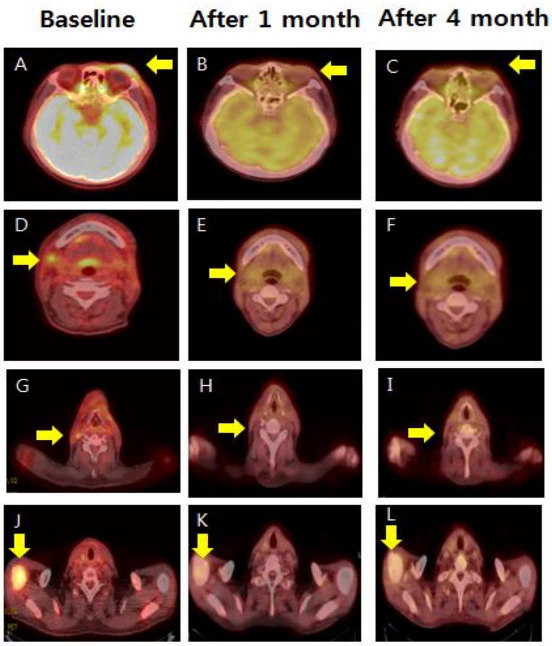


| | 2013-08-28 (Baseline) | 2013-11-14 (At 1 month) | 2014-02-18 (At 4 month) |
|---|---|---|---|
| **Left orbit** | 4.6 | 2.3 (-50%) | 2.14 (-53.5%) |
| **Rt humerus** | 4 | 2.9 (-27.5%) | 1.98 (-50.5%) |
| **Rt neck LN1** | 2.8 | 1.3 (-53.6%) | 1.11 (-60.3%) |
| **Rt neck LN2** | 3.5 | 1.5 (-57.1%) | 1.4 (-60%) |

**Reduction in $SUV_{max}$ in target lesions**

*Everolimus was started on Oct, 10th, 2013.

**Supplemenary Fig.S3.** Comparison of PET-CT taken at baseline, after 1 month and after 4 month which showed reduction of $SUV_{max}$ in left orbit **(A, B, C)**, right neck nodes **(D, E, F & G, H, I)** and right humerus **(J, K, L).** The patient showed partial response as per PET response criteria.

(A)

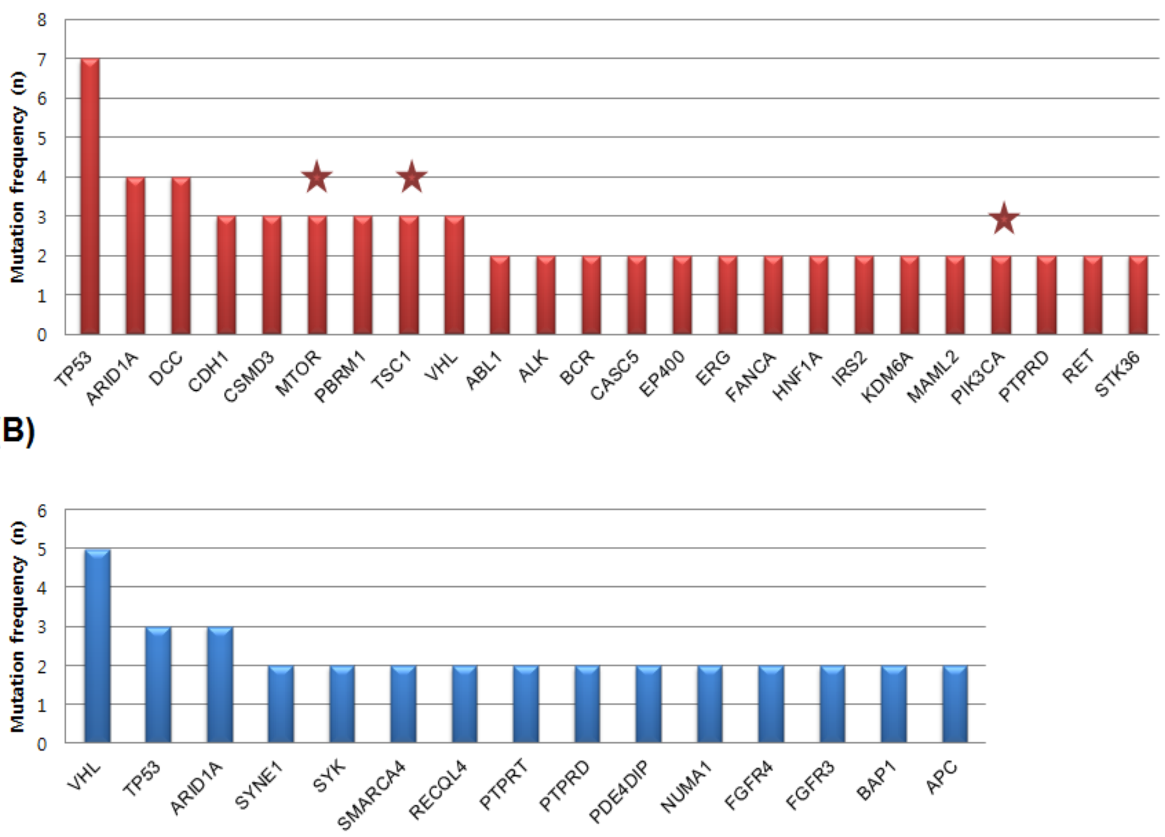


**Supplementary Fig.S4.** Recurrent genetic alterations seen in patients with **(A)** clinical benefit **(B)** non-clinical benefit

*MTOR*
p.K1771R (c.5312 A>G)

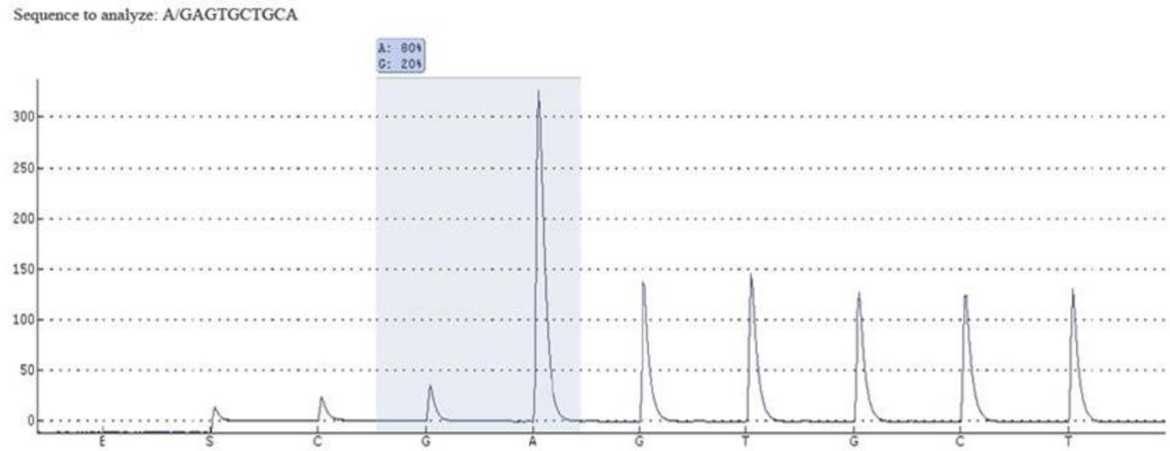


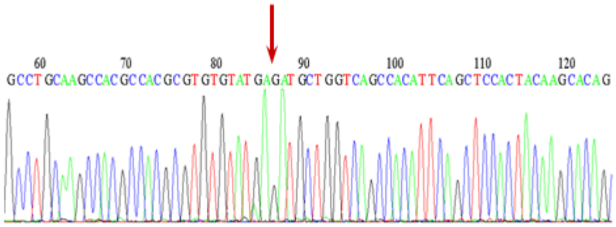

**#12** *TSC2*
p.E588K (c.1762 G>A)

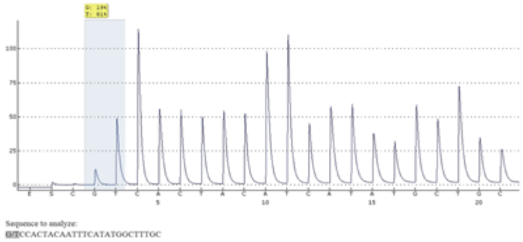

**#14** *NF1*
p.D1644A (c.4931 A>C)

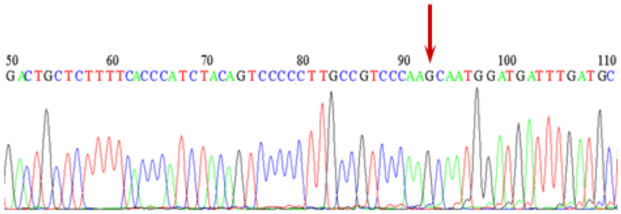

**#14** *TP53*
p.Q38fs (c.114 del A)

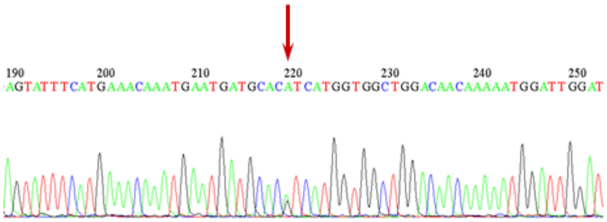

**#15** *PIK3CA*
p.H1047R (c.3140 A>G)

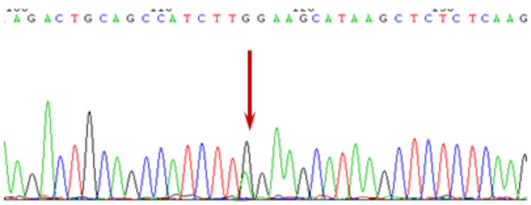

**#22** *TSC1*
p.W103X (c.308 G>A)

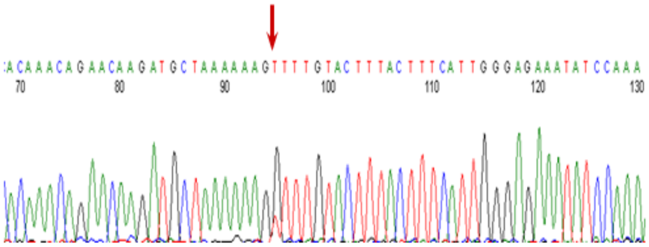

**#25** *PTEN*
c.801+1 G>T

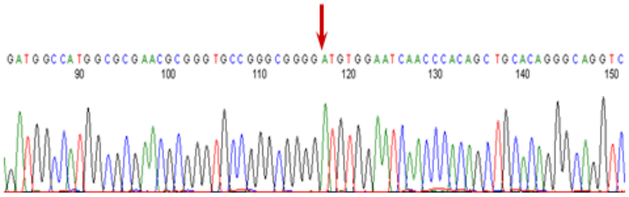

**#32** *TP53*
p.P19S (c.55 C>T)

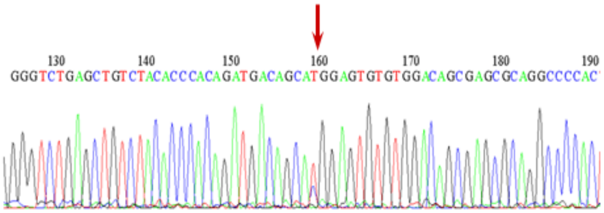

**#33** *AKT1*
p.M458T (c.1373 T>C)

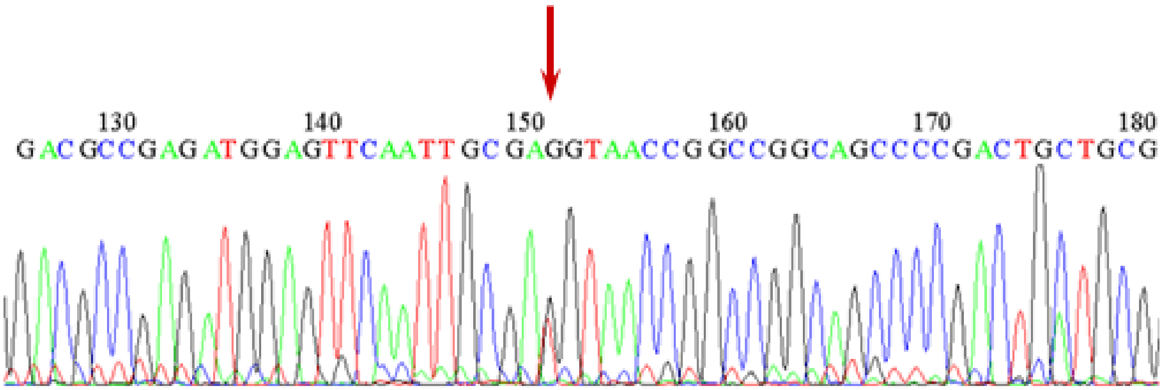


**#38** *NF2*
p.E38D (c.114 G>T)

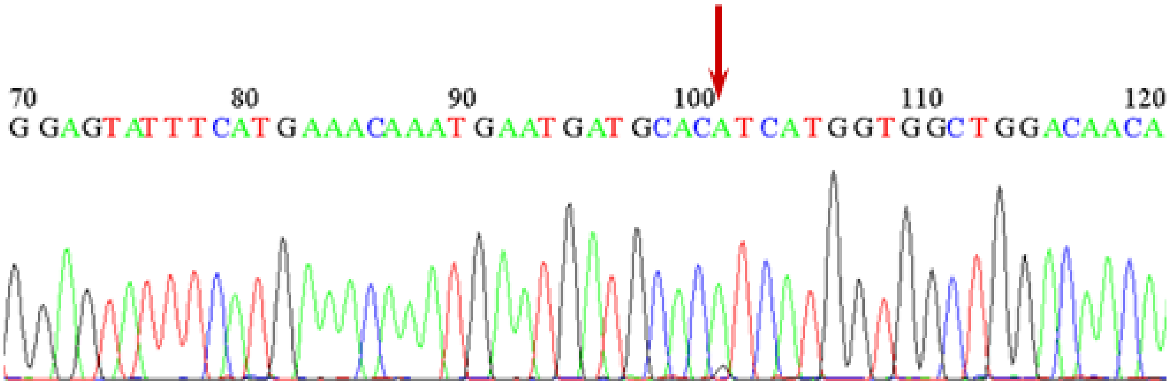


**#39** *PIK3CA*
p.H1047R (c.3140 A>G)

**Supplementary Fig.S5.** Validation of identified mutations in *MTOR, TSC1, TSC2, PIK3CA, NF1, NF2, PTEN, AKT1* by Sanger sequencing or pyrosequencing.
