# Supplementary material for: Next-generation sequencing reveals somatic mutations that confer exceptional response to everolimus
Source: Oncotarget. 2016 Feb 7;7(9):10547–56. doi: 10.18632/oncotarget.7234 (PMC4891139; doi:10.18632/oncotarget.7234)
Supplement: Supplementary file 2 [file oncotarget-07-10547-s002.pdf]

Supplementary table 1. Clinical trials participated by study populations

Hospital	Cancer type	Name of clinical trial	NCT ID	Regimen
Severance	Thyroid	A multicenter, phase II trial of everolimus in locally advanced or metastatic thyroid cancer of all histologic subtypes	NCT01164176	Everolimus
Severance	Stomach	Safety and efficacy of RAD001 (everolimus) monotherapy plus best supportive care in patients with advanced gastric cancer (GRANITE-1)	NCT00879333	Everolimus
Asan Medical Center	Stomach	Safety and efficacy of RAD001 (everolimus) monotherapy plus best supportive care in patients with advanced gastric cancer (GRANITE-1)	NCT00879333	Everolimus
Asan Medical Center	Stomach	RAD001 salvage monotherapy in advanced gastric cancer who failed standard first-line treatment	NCT00729482	Everolimus
Asan Medical Center	Stomach	RAD001 in advanced gastric cancer who failed standard first-line treatment with pS6 Ser 240/4 expression	NCT01482299	Everolimus
Samsung Medical Center	Stomach	Safety and efficacy of RAD001 (everolimus) monotherapy plus best supportive care in patients with advanced gastric cancer (GRANITE-1)	NCT00879333	Everolimus
Severance	Sarcoma	RAD001 in advanced sarcoma	NCT01830153	Everolimus
Asan Medical Center	Sarcoma	RAD001 in advanced sarcoma	NCT01830153	Everolimus
Samsung Medical Center	Sarcoma	RAD001 in advanced sarcoma	NCT01830153	Everolimus
Seoul National University	Sarcoma	RAD001 in advanced sarcoma	NCT01830153	Everolimus
Korea University	Sarcoma	RAD001 in advanced sarcoma	NCT01830153	Everolimus
Youngnam University	Sarcoma	RAD001 in advanced sarcoma	NCT01830153	Everolimus
Severance	Renal	N/A	N/A	Everolimus
Asan Medical Center	Renal	N/A	N/A	Everolimus

N/A: Not a clinical trial. Everolimus was used after approval by the Korean FDA as the second line of therapy in renal cell carcinoma

Supplementary table 2. Sanger and pyrosequencing primers for validation of mutations acquired from CCP panel

Number*	Gene	Chr	Loci	Forward	Reverse	Pyrosequencing primer
1	MTOR	chr1	11193189	AGCTGCGGTCGTGCTCTG	GAGTGGCAGCTGAATCTACAGGG	ATGAGAGCACAATCCCCA
4	PIK3CA	chr3	178936082	AGAGGGGAAAAATATGACA	GCATTTAATGTGCCAACTACC	
5	TSC1	chr9	135786839	CACACACTAACCCCCTGTGTT	TGAAATTTTCCCAACCACATACT	
10	MTOR	chr1	11188177	TGGTAGACTTAGAAGCCACTGTCA	TCATACCAGCTCTTCCCCAACC	TTTTCCCTCAGGCCC
12	TSC2	chr16	2120502	TGTTTTGAAGCACGCACTCT	GCTTCCAGGAACCACACCT	
14	NF1	chr17	29652933	CCATATTATGCAAAGCCATATGAA	TCTGTTTTAAAGCGATTGCTAGG	CCGGTATGGGTAAGG
14	TP53	chr17	7579573	GGTGTAGGAGCTGCTGGTG	CTGGTAAGGACAAGGGTTGG	
15	PIK3CA	chr3	178952085	ATGCTTGGCTCTGGAATGC	GGTCTTTGCCTGCTGAGAGT	
22	TSC1	chr9	135801029	TGCATGACCCTTGCTTTACA	GCCAAATGCCTAGAAGGTTTT	
25	PTEN	chr10	89717777	TGCAGATCCTCAGTTTGTGG	CACCAATGCCAGAGTAAGCA	
32	TP53	chr17	7578479	AACCAGCCCTGTCGTCTCT	GTTTCTTTGCTGCCGTCTTC	
33	AKT1	chr14	105236748	CTCAAATGCACCCGAGAAAT	CCAGGTCCCTGTGTCAATCT	
38	NF2	chr22	30000101	GGGCTAAAGGGCTCAGAGTG	TGAGGTTTTGGGCCTAGTTG	
39	PIK3CA	chr3	178952085	ATGCTTGGCTCTGGAATGC	GGTCTTTGCCTGCTGAGAGT	

* numbering was based on figure 2

** Mutations of MTOR and NF1 were validated by pyrosequencing

Supplementary table 3. Clinical outcomes of patients with clinical benefit (N=22)

Tumor site	Sex	Age	Tumor shrinkage (%)	Best response	PFS (mo)
Gastric	F	49	-64	PR	34.3
Gastric	F	71	-27	SD	3.8
Gastric	F	67	-25	SD	14.4
Gastric	M	54	-3	SD	5.8
Gastric	F	61	-11	SD	5.0
Gastric	M	74	-5	SD	11.3
Gastric	M	67	-60	PR	9.8
Gastric	F	73	-30	PR	7.2
Sarcoma	M	83	-47	PR	6.0
Sarcoma	M	34	3	SD	9.3
Sarcoma	M	47	-13	SD	20.3
Sarcoma	M	57	-24	SD	5.0
Thyroid	F	72	-21	SD	2.4
Thyroid	M	55	-3	SD	21.1
Kidney	M	65	-45	PR	22.1
Kidney	M	56	-34	PR	15.1
Kidney	M	50	-22	SD	39.7+
Kidney	F	76	-30	PR	14.3
Kidney	F	53	-5	SD	35.6+
Kidney	M	68	-9	SD	32.8
Head and neck	M	51	-34	PR	13
Head and neck	M	37	-30	PR	6

Supplementary table 4. Clinical outcomes of patients without clinical benefit (N=17)

Tumor site	Sex	Age	Tumor shrinkage (%)	Best response	PFS (mo)
Gastric	M	71	76	PD	1.7
Gastric	F	48	14, new lesion	PD	1.6
Gastric	M	72	30	PD	1.4
Gastric	M	58	3, new lesion	PD	3.6
Gastric	F	65	NE, new lesion	PD	0.6
Sarcoma	F	57	33	PD	1.6
Sarcoma	F	26	68	PD	0.9
Sarcoma	F	53	17, new lesion	PD	1.6
Kidney	F	67	22, new lesion	PD	0.9
Kidney	M	45	34	PD	0.9
Kidney	M	50	60	PD	1.6
Kidney	M	63	80	PD	1.5
Kidney	M	52	60	PD	2.3
Kidney	M	64	70	PD	2.9
Kidney	M	64	40	PD	3.2
Kidney	M	45	140	PD	3.8
Kidney	F	52	100	PD	3.9

Supplementary table 5. Overall genetic alterations found in this study

Number*	Cancer type	Gene	Chr	Loci	Ref	Alt	Protein change	Annotation	Provean Score	Provean prediction	SIFT Score	SIFT prediction
1	AGC	CDH1	chr16	68844172	G	T	nonsynonymous SNV	CDH1:NM_004360:exon6:c.G760T:p.D254Y	-8.32	Deleterious	0	Damaging
1	AGC	CDH1	chr16	68849480	T	-	frameshift deletion	CDH1:NM_004360:exon10:c.1383delT:p.P461fs				
1	AGC	CSMD3	chr8	114111056	T	C	nonsynonymous SNV	CSMD3:NM_052900:exon5:c.A846G:p.I282M	-1.05	Neutral	0.132	Tolerated
1	AGC	CSMD3	chr8	113988174	T	C	nonsynonymous SNV	CSMD3:NM_198123:exon7:c.A1234G:p.K412E	-0.13	Neutral	0.116	Tolerated
1	AGC	IKBKE	chr1	206651597	A	G	nonsynonymous SNV	IKBKE:NM_001193321:exon8:c.A652G:p.S218G	-2.38	Neutral	0.005	Damaging
1	AGC	KAT6B	chr10	76781848	C	G	nonsynonymous SNV	KAT6B:NM_001256468:exon16:c.C2682G:p.D894E	-0.1	Neutral	1	Tolerated
1	AGC	MTOR	chr1	11193189	T	C	nonsynonymous SNV	MTOR:NM_004958:exon38:c.A5312G:p.K1771R	-0.61	Neutral	0.307	Tolerated
1	AGC	TNK2	chr3	195595107	C	T	nonsynonymous SNV	TNK2:NM_005781:exon12:c.G2017A:p.V673M	-0.56	Neutral	0.05	Damaging
1	AGC	TPR	chr1	186291658	C	T	nonsynonymous SNV	TPR:NM_003292:exon44:c.G6358A:p.G2120S	-0.57	Neutral	0.104	Tolerated
2	RCC	ARID1A	chr1	27101708	A	C	nonsynonymous SNV	ARID1A:NM_006015:exon18:c.A4990C:p.I1664L	-1.64	Neutral	0.161	Tolerated
2	RCC	ARID1A	chr1	27097814	C	G	nonsynonymous SNV	ARID1A:NM_006015:exon12:c.C3403G:p.P1135A	-6.63	Deleterious	0	Damaging
2	RCC	MARK4	chr19	45790780	G	A	nonsynonymous SNV	MARK4:NM_001199867:exon13:c.G1352A:p.R451Q	-1.16	Neutral	0.227	Tolerated
2	RCC	NLRP1	chr17	5463364	C	G	splicing	NLRP1(NM_001033053:exon5:c.653-1G>C				
2	RCC	SYNE1	chr6	152730316	C	G	nonsynonymous SNV	SYNE1:NM_033071:exon44:c.G6448C:p.D2150H	-4.7	Deleterious	0.001	Damaging
2	RCC	TET1	chr10	70406352	C	T	nonsynonymous SNV	TET1:NM_030625:exon4:c.C3866T:p.T1289M	-1.11	Neutral	0.204	Tolerated
3	RCC	FANCJ	chr17	59934437	T	C	nonsynonymous SNV	BRIP1:NM_032043:exon4:c.A361G:p.T121A	-0.32	Neutral	0.719	Tolerated
3	RCC	PDE4DIP	chr1	144906085	C	-	frameshift deletion	PDE4DIP:NM_001002811:exon15:c.3037delG:p.E1013fs				
3	RCC	SETD2	chr3	47164553	C	A	stopgain SNV	SETD2:NM_014159:exon3:c.G1573T:p.E525X				
3	RCC	VHL	chr3	10191504	T	G	nonsynonymous SNV	VHL:NM_198156:exon2:c.T374G:p.V125G	-5.17	Deleterious	NA	NA
4	RCC	ABL1	chr9	1337303	G	A	nonsynonym	ABL1:NM_005157:exon3:c.G430A:p.G144R	-5.77	Deleteriou	0.111	Tolerated

				64			ous SNV			s		
4	RCC	LPHN3	chr4	62935919	G	A	nonsynonymous SNV	LPHN3:NM_015236:exon23:c.G3703A:p.E1235K	-2.5	Deleterious	0.003	Damaging
4	RCC	PIK3CA	chr3	178936082	G	A	nonsynonymous SNV	PIK3CA:NM_006218:exon10:c.G1624A:p.E542K	-2.09	Neutral	0.016	Damaging
5	RCC	BCL11A	chr2	60688772	C	G	nonsynonymous SNV	BCL11A:NM_018014:exon4:c.G1275C:p.K425N	-4.3	Deleterious	0.001	Damaging
5	RCC	TSC1	chr9	135786839	G	A	splicing	TSC1(NM_000368:exon11:c.1029+1G>A				
6	Thyroid	SGK1	chr6	134492277	T	C	nonsynonymous SNV	SGK1:NM_001143677:exon10:c.A1006G:p.N336D	-3.85	Deleterious	0.043	Damaging
7	Sarcoma	TRRAP	chr7	98528356	A	G	nonsynonymous SNV	TRRAP:NM_001244580:exon25:c.A3494G:p.K1165R	-1.62	Neutral	0.175	Tolerated
8	RCC	BAI3	chr6	69758142	C	T	nonsynonymous SNV	BAI3:NM_001704:exon14:c.C2173T:p.R725W	1.86	Neutral	0.422	Tolerated
8	RCC	BRD3	chr9	136916698	C	T	nonsynonymous SNV	BRD3:NM_007371:exon4:c.G485A:p.G162E	-1.86	Neutral	0.149	Tolerated
8	RCC	CCNE1	chr19	30313408	G	T	nonsynonymous SNV	CCNE1:NM_001238:exon11:c.G1008T:p.M336I	-1.34	Neutral	0.493	Tolerated
8	RCC	ERCC4	chr16	14041971	G	A	nonsynonymous SNV	ERCC4:NM_005236:exon11:c.G2518A:p.E840K	-0.99	Neutral	0.245	Tolerated
8	RCC	MYH11	chr16	15844183	G	A	nonsynonymous SNV	MYH11:NM_002474:exon16:c.C1870T:p.R624C	-4.14	Deleterious	0.003	Damaging
8	RCC	PBRM1	chr3	52668671	T	-	frameshift deletion	PBRM1:NM_018313:exon12:c.1248delA:p.K416fs				
8	RCC	STK36	chr2	219564031	T	C	nonsynonymous SNV	STK36:NM_001243313:exon26:c.T3701C:p.I1234T	-1.97	Neutral	0.003	Damaging
8	RCC	TET2	chr4	106156616	G	A	nonsynonymous SNV	TET2:NM_001127208:exon3:c.G1517A:p.R506K	-0.51	Neutral	0.11	Tolerated
8	RCC	USP9X	chrX	41084138	A	G	nonsynonymous SNV	USP9X:NM_001039590:exon40:c.A6895G:p.T2299A	-1.42	Neutral	0.374	Tolerated
8	RCC	VHL	chr3	10188202	C	G	nonsynonymous SNV	VHL:NM_000551:exon2:c.C345G:p.H115Q	-6.39	Deleterious	NA	NA
9	AGC	PTPRD	chr9	8486128	C	A	nonsynonymous SNV	PTPRD:NM_002839:exon28:c.G2689T:p.A897S	-2.26	Neutral	0.002	Damaging
10	RCC	ALK	chr2	30143054	G	T	nonsynonymous SNV	ALK:NM_004304:exon1:c.C472A:p.P158T	-0.68	Neutral	0.015	Damaging
10	RCC	ALK	chr2	30143052	G	-	frameshift deletion	ALK:NM_004304:exon1:c.474delC:p.P158fs				
10	RCC	ARID1A	chr1	27105820	A	T	stopgain SNV	ARID1A:NM_006015:exon20:c.A5431T:p.K1811X				
10	RCC	BCR	chr22	23634787	C	T	nonsynonymous SNV	BCR:NM_004327:exon15:c.C2842T:p.R948C	-5.63	Deleterious	0.01	Damaging
10	RCC	CEBPA	chr1	3379231	G	A	nonsynonym	CEBPA:NM_004364:exon1:c.C1010T:p.T337	-5.35	Deleteriou	0.006	Damagin

			9	1			ous SNV	M		s		g
10	RCC	FANCA	chr16	89871788	C	T	nonsynonymous SNV	FANCA:NM_000135:exon7:c.G609A:p.M203I	-0.32	Neutral	0.213	Tolerated
10	RCC	FANCA	chr16	89871787	G	A	nonsynonymous SNV	FANCA:NM_000135:exon7:c.C610T:p.H204Y	-1.2	Neutral	0.051	Tolerated
10	RCC	HNF1A	chr12	121434170	C	T	nonsynonymous SNV	HNF1A:NM_000545:exon5:c.C1061T:p.T354M	-0.21	Neutral	0.03	Damaging
10	RCC	MAML2	chr11	95826591	C	G	nonsynonymous SNV	MAML2:NM_032427:exon2:c.G604C:p.D202H	-2.79	Deleterious	0.099	Tolerated
10	RCC	MLL2	chr12	49445858	-	G	frameshift insertion	KMT2D:NM_003482:exon10:c.1607_1608insC:p.L536fs				
10	RCC	MTOR	chr1	11188177	T	A	nonsynonymous SNV	MTOR:NM_004958:exon43:c.A5917T:p.I1973F	-3.56	Deleterious	0.001	Damaging
10	RCC	NTRK3	chr15	88679826	T	A	nonsynonymous SNV	NTRK3:NM_001007156:exon8:c.A637T:p.S213C	-2.37	Neutral	0.036	Damaging
10	RCC	PBRM1	chr3	52598099	G	C	nonsynonymous SNV	PBRM1:NM_018313:exon24:c.C3767G:p.A1256G	-0.88	Neutral	0.305	Tolerated
10	RCC	STK36	chr2	219540963	C	A	nonsynonymous SNV	STK36:NM_001243313:exon6:c.C646A:p.P216T	-6.09	Deleterious	0.019	Damaging
10	RCC	VHL	chr3	10188256	T	-	frameshift deletion	VHL:NM_000551:exon2:c.399delT:p.T133fs				
12	AGC	NBN	chr8	90993101	A	T	nonsynonymous SNV	NBN:NM_002485:exon4:c.T341A:p.V114D	-6.13	Deleterious	0	Damaging
12	AGC	TP53	chr17	7577511	A	T	nonsynonymous SNV	TP53:NM_001126115:exon3:c.T374A:p.L125Q	-5.64	Deleterious	0	Damaging
12	AGC	TSC2	chr16	2120502	G	A	nonsynonymous SNV	TSC2:NM_000548:exon17:c.G1762A:p.E588K	-1.29	Neutral	0.588	Tolerated
13	Sarcoma	GRM8	chr7	126410077	C	G	nonsynonymous SNV	GRM8:NM_000845:exon6:c.G1199C:p.G400A	-3.9	Deleterious	0.002	Damaging
14	Orbital	HLF	chr17	53398125	C	G	nonsynonymous SNV	HLF:NM_002126:exon4:c.C773G:p.S258W	-4.52	Deleterious	0.001	Damaging
14	Orbital	NF1	chr17	29652933	A	C	nonsynonymous SNV	NF1:NM_001042492:exon37:c.A4931C:p.D1644A	-7.16	Deleterious	0	Damaging
14	Orbital	TP53	chr17	7579573	T	-	frameshift deletion	TP53:NM_000546:exon4:c.114delA:p.Q38fs				
15	Parotid	CSMD3	chr8	113418873	C	T	nonsynonymous SNV	CSMD3:NM_052900:exon34:c.G5377A:p.G1793S	-5.19	Deleterious	0.036	Damaging
15	Parotid	DCC	chr18	50592472	A	C	nonsynonymous SNV	DCC:NM_005215:exon7:c.A1197C:p.Q399H	-4.36	Deleterious	0	Damaging
15	Parotid	EML4	chr2	42483751	C	G	nonsynonymous SNV	EML4:NM_001145076:exon3:c.C319G:p.L107V	-0.09	Neutral	0.575	Tolerated
15	Parotid	ERCC1	chr19	45924573	C	A	stopgain SNV	ERCC1:NM_202001:exon2:c.G184T:p.E62X				
15	Parotid	IRS2	chr1	1104368	C	T	nonsynonym	IRS2:NM_003749:exon1:c.G1534A:p.A512T	-0.73	Neutral	0.393	Tolerated

			3	67			ous SNV					
15	Parotid	PIK3CA	chr3	178952085	A	G	nonsynonymous SNV	PIK3CA:NM_006218:exon21:c.A3140G:p.H1047R	-1.38	Neutral	0.019	Damaging
15	Parotid	SAMD9	chr7	92733765	A	G	nonsynonymous SNV	SAMD9:NM_001193307:exon2:c.T1646C:p.V549A	-1.57	Neutral	0.003	Damaging
15	Parotid	TP53	chr17	7578512	-	TC	frameshift insertion	TP53:NM_001126115:exon1:c.21_22insGA:p.T8fs				
15	Parotid	TSHR	chr14	81609604	A	C	nonsynonymous SNV	TSHR:NM_000369:exon10:c.A1202C:p.K401T	-1.64	Neutral	0.057	Tolerated
16	AGC	DAXX	chr6	33287886	T	C	nonsynonymous SNV	DAXX:NM_001254717:exon4:c.A1142G:p.E381G	-1.68	Neutral	0.006	Damaging
16	AGC	EGFR	chr7	55242440	A	C	nonsynonymous SNV	EGFR:NM_005228:exon19:c.A2210C:p.K737T	0.78	Neutral	0.747	Tolerated
16	AGC	MLL3	chr7	151900098	G	A	nonsynonymous SNV	KMT2C:NM_170606:exon26:c.C4013T:p.T1338I	-1.74	Neutral	0.153	Tolerated
16	AGC	RET	chr10	43595976	C	T	nonsynonymous SNV	RET:NM_020630:exon2:c.C143T:p.T48M	-1.53	Neutral	0.096	Tolerated
16	AGC	RNF213	chr17	78280074	A	G	nonsynonymous SNV	RNF213:NM_001256071:exon12:c.A2233G:p.R745G	-1.8	Neutral	0.239	Tolerated
16	AGC	TP53	chr17	7577154	C	-	frameshift deletion	TP53:NM_001126115:exon4:c.388delG:p.G130fs				
16	AGC	TP53	chr17	7577153	C	A	nonsynonymous SNV	TP53:NM_001126115:exon4:c.G389T:p.G130V	-7.92	Deleterious	0	Damaging
17	AGC	CASC5	chr15	40917282	A	G	nonsynonymous SNV	CASC5:NM_144508:exon10:c.A4820G:p.N1607S	-1.87	Neutral	0.144	Tolerated
17	AGC	CTNNB1	chr3	41266885	C	A	nonsynonymous SNV	CTNNB1:NM_001098209:exon5:c.C556A:p.H186N	-3.63	Deleterious	0.149	Tolerated
17	AGC	DCC	chr18	50961534	T	A	nonsynonymous SNV	DCC:NM_005215:exon22:c.T3184A:p.Y1062N	-1.41	Neutral	0.294	Tolerated
17	AGC	FLT1	chr13	29002013	C	A	nonsynonymous SNV	FLT1:NM_001159920:exon9:c.G1152T:p.L384F	-0.7	Neutral	0.467	Tolerated
17	AGC	KDR	chr4	55972919	A	C	nonsynonymous SNV	KDR:NM_002253:exon11:c.T1471G:p.F491V	-0.47	Neutral	0.292	Tolerated
17	AGC	LTK	chr15	41800275	G	A	nonsynonymous SNV	LTK:NM_001135685:exon8:c.C1058T:p.T353I	-3.93	Deleterious	0.001	Damaging
17	AGC	MTRR	chr5	7886757	G	C	nonsynonymous SNV	MTRR:NM_002454:exon8:c.G1087C:p.G363R	-0.06	Neutral	0.606	Tolerated
17	AGC	TP53	chr17	7577506	C	A	nonsynonymous SNV	TP53:NM_001126115:exon3:c.G379T:p.D127Y	-1.89	Neutral	0.025	Damaging
17	AGC	TRIP11	chr14	92477374	T	C	nonsynonymous SNV	TRIP11:NM_004239:exon9:c.A1270G:p.I424V	-0.05	Neutral	1	Tolerated
18	Sarcoma	ABL1	chr9	133760495	G	C	nonsynonymous SNV	ABL1:NM_005157:exon11:c.G2818C:p.A940P	0.35	Neutral	0.085	Tolerated
18	Sarco	ABL2	chr1	1790775	T	C	nonsynonym	ABL2:NM_001168236:exon11:c.A2740G:p.I9	0.08	Neutral	0.008	Damagin

	ma			99			ous SNV	14V				g
18	Sarcoma	BCL6	chr3	187447245	G	T	nonsynonymous SNV	BCL6:NM_001134738:exon4:c.C948A:p.F316L	-1.48	Neutral	0.075	Tolerated
18	Sarcoma	CARD11	chr7	2959049	T	C	nonsynonymous SNV	CARD11:NM_032415:exon18:c.A2467G:p.T823A	-2.93	Deleterious	0.005	Damaging
18	Sarcoma	CDH20	chr18	59170324	T	C	nonsynonymous SNV	CDH20:NM_031891:exon4:c.T800C:p.V267A	-3.82	Deleterious	0	Damaging
18	Sarcoma	EP400	chr12	132516635	T	G	nonsynonymous SNV	EP400:NM_015409:exon30:c.T5892G:p.D1964E	-3.48	Deleterious	0.007	Damaging
18	Sarcoma	EP400	chr12	132512030	C	A	nonsynonymous SNV	EP400:NM_015409:exon26:c.C5063A:p.P1688H	-1.56	Neutral	0.004	Damaging
18	Sarcoma	ERG	chr21	39755427	AA	-	frameshift deletion	ERG:NM_001136155:exon9:c.1061_1062del:p.354_354del				
18	Sarcoma	ERG	chr21	39755432	A	T	nonsynonymous SNV	ERG:NM_001136155:exon9:c.T1057A:p.F353I	-2.4	Neutral	0.08	Tolerated
18	Sarcoma	HNF1A	chr12	121435466	A	G	nonsynonymous SNV	HNF1A:NM_000545:exon7:c.A1499G:p.H500R	-3.05	Deleterious	0.014	Damaging
18	Sarcoma	IGF1R	chr15	99250824	A	G	nonsynonymous SNV	IGF1R:NM_000875:exon2:c.A128G:p.Y43C	0.01	Neutral	0.001	Damaging
18	Sarcoma	IKZF1	chr7	50459454	A	G	nonsynonymous SNV	IKZF1:NM_001220775:exon3:c.A68G:p.E23G	-3.08	Deleterious	0.075	Tolerated
18	Sarcoma	IRS2	chr13	110436867	C	T	nonsynonymous SNV	IRS2:NM_003749:exon1:c.G1534A:p.A512T	-0.73	Neutral	0.393	Tolerated
18	Sarcoma	KDM5C	chrX	53222696	C	A	nonsynonymous SNV	KDM5C:NM_001146702:exon23:c.G4030T:p.D1344Y	-5.86	Deleterious	0.003	Damaging
18	Sarcoma	MAML2	chr11	96074609	T	C	nonsynonymous SNV	MAML2:NM_032427:exon1:c.A451G:p.N151D	-1.26	Neutral	0.343	Tolerated
18	Sarcoma	MTOR	chr1	11227567	T	C	nonsynonymous SNV	MTOR:NM_004958:exon29:c.A4261G:p.N1421D	-4.05	Deleterious	0.004	Damaging
18	Sarcoma	MYCL1	chr1	40363136	C	A	nonsynonymous SNV	MYCL1:NM_001033081:exon2:c.G1003T:p.G335W	-2.83	Deleterious	0.005	Damaging
18	Sarcoma	NCOA2	chr8	71069293	A	G	nonsynonymous SNV	NCOA2:NM_006540:exon11:c.T1307C:p.M436T	-0.95	Neutral	0.319	Tolerated
18	Sarcoma	NUMA1	chr11	71717131	C	T	nonsynonymous SNV	NUMA1:NM_006185:exon22:c.G5642A:p.R1881H	-1.79	Neutral	0	Damaging
18	Sarcoma	PAK3	chrX	110390984	C	T	nonsynonymous SNV	PAK3:NM_001128167:exon4:c.C296T:p.A99V	-3.39	Deleterious	0.028	Damaging
18	Sarcoma	POT1	chr7	124503601	G	A	nonsynonymous SNV	POT1:NM_015450:exon8:c.C349T:p.R117C	-4.52	Deleterious	0.001	Damaging
19	Sarcoma	AR	chrX	66942788	T	C	nonsynonymous SNV	AR:NM_000044:exon7:c.T2569C:p.F857L	-4.36	Deleterious	0.06	Tolerated
19	Sarcoma	ARID1A	chr1	27089568	C	G	nonsynonymous SNV	ARID1A:NM_006015:exon8:c.C2524G:p.H842D	-4.57	Deleterious	0.006	Damaging
19	Sarco	ATRX	chr	7685521	C	T	nonsynonym	ATRX:NM_138270:exon23:c.G5662A:p.D188	-2.58	Deleteriou	0.026	Damagin

	ma		X	1			ous SNV	8N		s		g
19	Sarcoma	CASC5	chr15	40937970	G	T	nonsynonymous SNV	CASC5:NM_144508:exon16:c.G6006T:p.Q2002H	-2.76	Deleterious	0.005	Damaging
19	Sarcoma	DCC	chr18	50923793	C	G	nonsynonymous SNV	DCC:NM_005215:exon18:c.C2804G:p.A935G	-3.46	Deleterious	0.001	Damaging
19	Sarcoma	DCC	chr18	50731684	G	A	nonsynonymous SNV	DCC:NM_005215:exon10:c.G1672A:p.V558I	0.25	Neutral	1	Tolerated
19	Sarcoma	FANCD2	chr3	10074658	T	A	splicing	FANCD2(NM_033084:exon3:c.205+2T>A				
19	Sarcoma	GATA1	chrX	48651607	G	A	nonsynonymous SNV	GATA1:NM_002049:exon5:c.G773A:p.C258Y	-10.02	Deleterious	0	Damaging
19	Sarcoma	KDM6A	chrX	44969324	G	A	nonsynonymous SNV	KDM6A:NM_021140:exon28:c.G4006A:p.V1336M	-1.79	Neutral	0.001	Damaging
19	Sarcoma	KDM6A	chrX	44820554	T	C	nonsynonymous SNV	KDM6A:NM_021140:exon3:c.T251C:p.I84T	-4.37	Deleterious	0.004	Damaging
19	Sarcoma	MLLT10	chr10	21884295	G	A	nonsynonymous SNV	MLLT10:NM_001195626:exon4:c.G331A:p.E111K	-3.27	Deleterious	0.001	Damaging
19	Sarcoma	PARP1	chr1	226566955	C	A	nonsynonymous SNV	PARP1:NM_001618:exon12:c.G1633T:p.V545F	-4.39	Deleterious	0.001	Damaging
19	Sarcoma	PIK3CG	chr7	106520102	A	G	nonsynonymous SNV	PIK3CG:NM_002649:exon6:c.A2530G:p.I844V	0.46	Neutral	0.381	Tolerated
19	Sarcoma	PTPRD	chr9	8465570	T	C	nonsynonymous SNV	PTPRD:NM_001171025:exon14:c.A2347G:p.T783A	-2.49	Neutral	0.546	Tolerated
19	Sarcoma	RET	chr10	43617412	T	C	nonsynonymous SNV	RET:NM_020630:exon16:c.T2749C:p.W917R	-13	Deleterious	0	Damaging
19	Sarcoma	SMAD2	chr18	45372065	C	A	nonsynonymous SNV	SMAD2:NM_001135937:exon8:c.G1014T:p.W338C	-8.5	Deleterious	0.06	Tolerated
19	Sarcoma	TAF1	chrX	70603959	A	G	nonsynonymous SNV	TAF1:NM_004606:exon13:c.A2155G:p.T719A	-2.21	Neutral	0.165	Tolerated
19	Sarcoma	TRIM33	chr1	115006163	C	T	nonsynonymous SNV	TRIM33:NM_015906:exon3:c.G661A:p.E221K	-2.32	Neutral	0.147	Tolerated
19	Sarcoma	TSC1	chr9	135786949	G	A	nonsynonymous SNV	TSC1:NM_000368:exon10:c.C920T:p.A307V	-0.57	Neutral	0.14	Tolerated
20	AGC	PAX3	chr2	223066132	C	T	nonsynonymous SNV	PAX3:NM_001127366:exon9:c.G1447A:p.A483T	-0.47	Neutral	0.014	Damaging
20	AGC	TP53	chr17	7577570	C	T	nonsynonymous SNV	TP53:NM_001126115:exon3:c.G315A:p.M105I	-3.89	Deleterious	0.007	Damaging
21	AGC	ATM	chr11	108165711	G	T	stopgain SNV	ATM:NM_000051:exon32:c.G4834T:p.E1612X				
21	AGC	BCR	chr22	23523848	G	A	nonsynonymous SNV	BCR:NM_004327:exon1:c.G701A:p.R234H	-1.55	Neutral	0.06	Tolerated
21	AGC	CDH1	chr16	68842678	T	C	nonsynonymous SNV	CDH1:NM_004360:exon5:c.T614C:p.F205S	-7.45	Deleterious	0	Damaging
21	AGC	ERBB2	chr1	3786635	G	A	nonsynonym	ERBB2:NM_004448:exon6:c.G661A:p.A221	-3.1	Deleteriou	0.048	Damagin

			7	6			ous SNV	T		s		g
21	AGC	LRP1B	chr2	141283417	G	T	stopgain SNV	LRP1B:NM_018557:exon49:c.C8022A:p.C2674X				
21	AGC	PBRM1	chr3	52643772	T	A	nonsynonymous SNV	PBRM1:NM_018313:exon17:c.A2124T:p.K708N	-3.48	Deleterious	0.004	Damaging
22	Thyroid	TSC1	chr9	135801029	G	A	stopgain SNV	TSC1:NM_000368:exon5:c.G308A:p.W103X				
23	RCC	BAP1	chr3	52443600	T	C	nonsynonymous SNV	BAP1:NM_004656:exon3:c.T92G:p.I31R	-6.6	Deleterious	0	Damaging
24	RCC	VHL	chr3	10188322	T	A	splicing	VHL(NM_000551:exon2:c.463+2T>A)				
25	RCC	BCL9	chr1	147092243	A	G	nonsynonymous SNV	BCL9:NM_004326:exon8:c.A2282G:p.Q761R	-0.62	Neutral	0.155	Tolerated
25	RCC	ERCC1	chr19	45926588	C	-	frameshift deletion	ERCC1:NM_202001:exon1:c.45delG:p.G15fs				
25	RCC	FGFR4	chr5	176520735	G	A	nonsynonymous SNV	FGFR4:NM_022963:exon9:c.G1358A:p.R453Q	-1.45	Neutral	0.097	Tolerated
25	RCC	LTF	chr3	46490484	C	A	nonsynonymous SNV	LTF:NM_001199149:exon9:c.G950T:p.R317L	-2.09	Neutral	0.251	Tolerated
25	RCC	MYCN	chr2	16082320	CG	-	frameshift deletion	MYCN:NM_005378:exon2:c.134_135del:p.45_45del				
25	RCC	PBRM1	chr3	52610581	T	-	frameshift deletion	PBRM1:NM_018313:exon23:c.3592delA:p.T1198fs				
25	RCC	PDE4DIP	chr1	144882471	G	A	nonsynonymous SNV	PDE4DIP:NM_001198834:exon24:c.C3548T:p.A1183V	-1.11	Neutral	0.016	Damaging
25	RCC	PTEN	chr10	89717777	G	T	splicing	PTEN(NM_000314:exon7:c.801+1G>T)				
25	RCC	SYK	chr9	93636506	C	G	nonsynonymous SNV	SYK:NM_001135052:exon7:c.C867G:p.N289K	-1.35	Neutral	0.808	Tolerated
25	RCC	VHL	chr3	10191507	GGAGCCT	-	frameshift deletion	VHL:NM_198156:exon2:c.377_383del:p.126_128del				
26	RCC	APC	chr5	112178913	T	C	nonsynonymous SNV	APC:NM_001127511:exon14:c.T7568C:p.I2523T	-0.81	Neutral	0.004	Damaging
26	RCC	FH	chr1	241661189	T	C	nonsynonymous SNV	FH:NM_000143:exon10:c.A1472G:p.Y491C	-3.44	Deleterious	0.023	Damaging
26	RCC	NUMA1	chr11	71724044	G	T	nonsynonymous SNV	NUMA1:NM_006185:exon15:c.C4505A:p.A1502D	-1.95	Neutral	0.005	Damaging
26	RCC	POT1	chr7	124503532	C	G	nonsynonymous SNV	POT1:NM_001042594:exon7:c.G25C:p.A9P	-3.3	Deleterious	0.008	Damaging
26	RCC	RET	chr10	43600475	G	T	nonsynonymous SNV	RET:NM_020630:exon4:c.G701T:p.R234L	-0.74	Neutral	0.125	Tolerated
26	RCC	VHL	chr3	10188200	C	A	nonsynonymous SNV	VHL:NM_000551:exon2:c.C343A:p.H115N	-5.5	Deleterious	NA	NA

27	RCC	SYNE1	chr6	152605301	T	C	nonsynonymous SNV	SYNE1:NM_033071:exon95:c.A17806G:p.M5936V	-1.65	Neutral	0.045	Damaging
27	RCC	VHL	chr3	10191540	T	C	nonsynonymous SNV	VHL:NM_198156:exon2:c.T410C:p.L137P	-5.43	Deleterious	NA	NA
28	Sarcoma	AR	chrX	66766196	C	T	nonsynonymous SNV	AR:NM_000044:exon1:c.C1208T:p.A403V	-1.26	Neutral	0.002	Damaging
28	Sarcoma	AXL	chr19	41726637	A	C	nonsynonymous SNV	AXL:NM_001699:exon2:c.A182C:p.Q61P	0.29	Neutral	0.409	Tolerated
28	Sarcoma	NCOA4	chr10	51584763	G	C	nonsynonymous SNV	NCOA4:NM_001145262:exon8:c.G862C:p.V288L	-1.66	Neutral	0.227	Tolerated
28	Sarcoma	NUMA1	chr11	71726012	C	A	nonsynonymous SNV	NUMA1:NM_006185:exon15:c.G2537T:p.R846L	-1.64	Neutral	0.688	Tolerated
28	Sarcoma	PKHD1	chr6	51503697	A	-	stopgain SNV	PKHD1:NM_138694:exon64:c.11456delT:p.L3819X				
28	Sarcoma	RECQL4	chr8	145741388	C	G	nonsynonymous SNV	RECQL4:NM_004260:exon5:c.G1115C:p.R372T				
28	Sarcoma	SMARCA4	chr19	11113760	A	G	nonsynonymous SNV	SMARCA4:NM_001128845:exon11:c.A1868G:p.E623G	-2.02	Neutral	0.217	Tolerated
28	Sarcoma	TP53	chr17	7578197	C	T	nonsynonymous SNV	TP53:NM_001126115:exon2:c.G256A:p.V86M	-2.57	Deleterious	0	Damaging
28	Sarcoma	TPR	chr1	186287919	C	A	stopgain SNV	TPR:NM_003292:exon47:c.G6610T:p.E2204X				
29	Sarcoma	BIRC3	chr11	102195462	T	A	nonsynonymous SNV	BIRC3:NM_001165:exon2:c.T222A:p.D74E	-1.65	Neutral	0.283	Tolerated
29	Sarcoma	RNF213	chr17	78337584	A	-	frameshift deletion	RNF213:NM_001256071:exon41:c.11744delA:p.E3915fs				
29	Sarcoma	TET2	chr4	106157284	C	G	nonsynonymous SNV	TET2:NM_001127208:exon3:c.C2185G:p.Q729E	-1.1	Neutral	0.003	Damaging
30	AGC	BTK	chrX	100612495	A	G	splicing	BTK(NM_000061:exon14:c.1177+2T>C)				
30	AGC	TP53	chr17	7577538	C	T	nonsynonymous SNV	TP53:NM_001126115:exon3:c.G347A:p.R116Q	-3.92	Deleterious	0.006	Damaging
30	AGC	WAS	chrX	48547108	A	C	nonsynonymous SNV	WAS:NM_000377:exon10:c.A991C:p.I331L	-0.05	Neutral	0.412	Tolerated
31	RCC	ADAMTS20	chr12	43822246	G	A	nonsynonymous SNV	ADAMTS20:NM_025003:exon26:c.C3743T:p.P1248L	-5.96	Deleterious	0.194	Tolerated
31	RCC	ARID1A	chr1	27088801	G	T	nonsynonymous SNV	ARID1A:NM_006015:exon7:c.G2410T:p.G804C	-7.08	Deleterious	0.001	Damaging
31	RCC	BAP1	chr3	52442023	C	-	frameshift deletion	BAP1:NM_004656:exon5:c.326delG:p.G109fs				
31	RCC	KDM5C	chrX	53223018	C	A	stopgain SNV	KDM5C:NM_001146702:exon22:c.G3853T:p.E1285X				
31	RCC	MALT1	chr18	56390370	C	-	frameshift deletion	MALT1:NM_173844:exon9:c.1076delC:p.T359fs				

31	RCC	MET	chr7	116340100	A	G	nonsynonymous SNV	MET:NM_000245:exon2:c.A962G:p.Y321C	-5.41	Deleterious	0.003	Damaging
31	RCC	PTPRT	chr20	40730802	C	T	nonsynonymous SNV	PTPRT:NM_007050:exon26:c.G3676A:p.G1226R	-5.66	Deleterious	0.001	Damaging
31	RCC	ROS1	chr6	117709188	A	G	nonsynonymous SNV	ROS1:NM_002944:exon13:c.T1769C:p.L590P	-0.07	Neutral	0.08	Tolerated
31	RCC	SAMD9	chr7	92734615	G	T	nonsynonymous SNV	SAMD9:NM_001193307:exon2:c.C796A:p.L266M	0.11	Neutral	0.221	Tolerated
31	RCC	SYNE1	chr6	152647441	G	T	nonsynonymous SNV	SYNE1:NM_033071:exon78:c.C15070A:p.L5024I	-0.19	Neutral	0.28	Tolerated
31	RCC	VHL	chr3	10188198	G	C	nonsynonymous SNV	VHL:NM_000551:exon2:c.G341C:p.G114A	-2.72	Deleterious	NA	NA
32	AGC	AKAP9	chr7	91726907	C	T	nonsynonymous SNV	AKAP9:NM_005751:exon42:c.C10406T:p.T3469M	-1.82	Neutral	0.006	Damaging
32	AGC	APC	chr5	112178441	T	A	nonsynonymous SNV	APC:NM_001127511:exon14:c.T7096A:p.L2366I	-0.5	Neutral	0.124	Tolerated
32	AGC	BCR	chr22	23596147	G	T	nonsynonymous SNV	BCR:NM_004327:exon2:c.G1441T:p.A481S	0.22	Neutral	1	Tolerated
32	AGC	FAM123B	chrX	63413082	C	-	frameshift deletion	AMER1:NM_152424:exon2:c.85delG:p.A29fs				
32	AGC	LPHN3	chr4	62903473	C	G	nonsynonymous SNV	LPHN3:NM_015236:exon21:c.C3412G:p.L1138V	-2.07	Neutral	0.163	Tolerated
32	AGC	PDE4DIP	chr1	144930732	G	C	nonsynonymous SNV	PDE4DIP:NM_001002811:exon1:c.C977G:p.P326R	-0.71	Neutral	0.476	Tolerated
32	AGC	PTPRD	chr9	8484252	C	T	nonsynonymous SNV	PTPRD:NM_001171025:exon12:c.G2017A:p.G673R	-6.61	Deleterious	0.032	Damaging
32	AGC	PTPRD	chr9	8465551	T	C	nonsynonymous SNV	PTPRD:NM_001171025:exon14:c.A2366G:p.H789R	-0.49	Neutral	0.525	Tolerated
32	AGC	PTPRT	chr20	40980792	G	T	nonsynonymous SNV	PTPRT:NM_007050:exon10:c.C1694A:p.T565N	-1.64	Neutral	0.032	Damaging
32	AGC	SYK	chr9	93641120	A	G	nonsynonymous SNV	SYK:NM_001135052:exon10:c.A1397G:p.N466S	-2.8	Deleterious	0.005	Damaging
32	AGC	TP53	chr17	7578479	G	A	nonsynonymous SNV	TP53:NM_001126115:exon1:c.C55T:p.P19S	-7.13	Deleterious	0.003	Damaging
33	AGC	AFF1	chr4	88036399	G	A	nonsynonymous SNV	AFF1:NM_005935:exon11:c.G2393A:p.S798N	-0.41	Neutral	0.049	Damaging
33	AGC	AKT1	chr14	105236748	A	G	nonsynonymous SNV	AKT1:NM_001014431:exon14:c.T1373C:p.M458T	-2.62	Deleterious	0.08	Tolerated
33	AGC	ARID1A	chr1	27057949	C	A	nonsynonymous SNV	ARID1A:NM_006015:exon3:c.C1657A:p.Q553K	-1.26	Neutral	0	Damaging
33	AGC	ARID2	chr12	46231105	T	-	frameshift deletion	ARID2:NM_152641:exon9:c.1025delT:p.L342fs				
33	AGC	EP300	chr22	41513641	A	G	nonsynonymous SNV	EP300:NM_001429:exon2:c.A545G:p.N182S	-0.66	Neutral	1	Tolerated

33	AGC	GRM8	chr7	126173205	A	C	nonsynonymous SNV	GRM8:NM_000845:exon8:c.T2231G:p.I744S	-4.51	Deleterious	0.105	Tolerated
33	AGC	PPARG	chr3	12475400	G	A	nonsynonymous SNV	PPARG:NM_005037:exon7:c.G1190A:p.R397H	-3.74	Deleterious	0	Damaging
33	AGC	RALGDS	chr9	135976951	C	T	nonsynonymous SNV	RALGDS:NM_001042368:exon16:c.G2245A:p.V749I	-0.43	Neutral	0.229	Tolerated
33	AGC	RECQL4	chr8	145738967	G	T	nonsynonymous SNV	RECQL4:NM_004260:exon13:c.C2188A:p.P730T				
33	AGC	SMO	chr7	128850922	G	C	nonsynonymous SNV	SMO:NM_005631:exon10:c.G1769C:p.S590T	-1.49	Neutral	0.219	Tolerated
34	RCC	PTCH1	chr9	98229671	C	T	nonsynonymous SNV	PTCH1:NM_000264:exon15:c.G2287A:p.V763I	-0.17	Neutral	0.59	Tolerated
34	RCC	SETD2	chr3	47163374	-	T	frameshift insertion	SETD2:NM_014159:exon3:c.2751_2752insA:p.S918fs				
35	RCC	CRBN	chr3	3197913	A	G	nonsynonymous SNV	CRBN:NM_001173482:exon6:c.T737C:p.L246S	-1.19	Neutral	0.055	Tolerated
36	RCC	BCL2L2	chr14	23778116	C	T	nonsynonymous SNV	BCL2L2:NM_001199839:exon4:c.C524T:p.T175M	-0.63	Neutral	0.058	Tolerated
36	RCC	CYP2D6	chr22	42525182	A	T	nonsynonymous SNV	CYP2D6:NM_000106:exon3:c.T358A:p.F120I	1.08	Neutral	0.948	Tolerated
36	RCC	EP400	chr12	132546686	T	G	nonsynonymous SNV	EP400:NM_015409:exon46:c.T7916G:p.V2639G	-3.78	Deleterious	0.001	Damaging
36	RCC	FGFR4	chr5	176524526	A	G	splicing	FGFR4(NM_002011:exon18:c.2260-2A>G				
36	RCC	MLL3	chr7	151878109	-	G	frameshift insertion	KMT2C:NM_170606:exon36:c.6835_6836insC:p.P2279fs				
36	RCC	TNK2	chr3	195594795	G	A	nonsynonymous SNV	TNK2:NM_005781:exon12:c.C2329T:p.R777W	-1.7	Neutral	0.001	Damaging
36	RCC	UGT1A1	chr2	234669549	T	C	nonsynonymous SNV	UGT1A1:NM_000463:exon1:c.T616C:p.F206L	-5.33	Deleterious	0.042	Damaging
38	AGC	ERBB3	chr12	56486844	G	A	nonsynonymous SNV	ERBB3:NM_001982:exon11:c.G1258A:p.G420S	-5.75	Deleterious	0.001	Damaging
38	AGC	FGFR3	chr4	1805554	G	C	nonsynonymous SNV	FGFR3:NM_000142:exon8:c.G1066C:p.V356L	-2.38	Neutral	0.001	Damaging
38	AGC	FGFR3	chr4	1805558	T	C	nonsynonymous SNV	FGFR3:NM_000142:exon8:c.T1070C:p.L357P	-3.43	Deleterious	0.015	Damaging
38	AGC	IGF1R	chr15	99434800	T	C	nonsynonymous SNV	IGF1R:NM_000875:exon3:c.T887C:p.F296S	-4.3	Deleterious	0.004	Damaging
38	AGC	KAT6A	chr8	41794836	T	C	nonsynonymous SNV	KAT6A:NM_006766:exon16:c.A3290G:p.Q1097R	-0.33	Neutral	0.437	Tolerated
38	AGC	NF2	chr22	30000101	G	T	nonsynonymous SNV	NF2:NM_000268:exon1:c.G114T:p.E38D	-2.12	Neutral	0.05	Damaging
38	AGC	SMARCA4	chr19	11141427	G	A	nonsynonymous SNV	SMARCA4:NM_001128845:exon24:c.G3404A:p.R1135Q	-3.77	Deleterious	0	Damaging

39	AGC	ARID1A	chr1	27106396	-	A	frameshift insertion	ARID1A:NM_006015:exon20:c.6007_6008insA:p.K2003fs				
39	AGC	PIK3CA	chr3	178952085	A	G	nonsynonymous SNV	PIK3CA:NM_006218:exon21:c.A3140G:p.H1047R	-1.38	Neutral	0.019	Damaging

Supplementary table 6. Validated alterations using sanger sequencing or pyrosequencing

Number*	Cancer type	Gene	Chr	Loci	Ref	Alt	Protein change	Annotation	Provean Score	Provean prediction	SIFT Score	SIFT prediction
1	AGC	MTOR	chr1	11193189	T	C	nonsynonymous SNV	MTOR:NM_004958:exon38:c.A5312G:p.K1771R	-0.61	Neutral	0.307	Tolerated
4	RCC	PIK3CA	chr3	178936082	G	A	nonsynonymous SNV	PIK3CA:NM_006218:exon10:c.G1624A:p.E542K	-2.09	Neutral	0.016	Damaging
5	RCC	TSC1	chr9	135786839	G	A	splicing	TSC1(NM_000368:exon11:c.1029+1G>A				
10	RCC	MTOR	chr1	11188177	T	A	nonsynonymous SNV	MTOR:NM_004958:exon43:c.A5917T:p.I1973F	-3.56	Deleterious	0.001	Damaging
12	AGC	TSC2	chr16	2120502	G	A	nonsynonymous SNV	TSC2:NM_000548:exon17:c.G1762A:p.E588K	-1.29	Neutral	0.588	Tolerated
14	Orbital	NF1	chr17	29652933	A	C	nonsynonymous SNV	NF1:NM_001042492:exon37:c.A4931C:p.D1644A	-7.16	Deleterious	0	Damaging
14	Orbital	TP53	chr17	7579573	T	-	frameshift deletion	TP53:NM_000546:exon4:c.114delA:p.Q38fs				
15	Parotid	PIK3CA	chr3	178952085	A	G	nonsynonymous SNV	PIK3CA:NM_006218:exon21:c.A3140G:p.H1047R	-1.38	Neutral	0.019	Damaging
22	Thyroid	TSC1	chr9	135801029	G	A	stopgain SNV	TSC1:NM_000368:exon5:c.G308A:p.W103X				
25	RCC	PTEN	chr10	89717777	G	T	splicing	PTEN(NM_000314:exon7:c.801+1G>T)				
32	AGC	TP53	chr17	7578479	G	A	nonsynonymous SNV	TP53:NM_001126115:exon1:c.C55T:p.P19S	-7.13	Deleterious	0.003	Damaging
33	AGC	AKT1	chr14	105236748	A	G	nonsynonymous SNV	AKT1:NM_001014431:exon14:c.T1373C:p.M458T	-2.62	Deleterious	0.08	Tolerated
38	AGC	NF2	chr22	30000101	G	T	nonsynonymous SNV	NF2:NM_000268:exon1:c.G114T:p.E38D	-2.12	Neutral	0.05	Damaging
39	AGC	PIK3CA	chr3	178952085	A	G	nonsynonymous SNV	PIK3CA:NM_006218:exon21:c.A3140G:p.H1047R	-1.38	Neutral	0.019	Damaging

*Mutaions of MTOR and NF1 were validated by pyrosequencing
